# Evaluation of Beneficial Influence of Local Application of *Crocus Pallasii Subsp. Haussknechtii Boiss*. Extract on Healing of Full Thickness Excisional Infected Wounds in Diabetic Rats

**DOI:** 10.30476/BEAT.2020.82567

**Published:** 2020-07

**Authors:** Mohammad Ali Ashja Zadeh, Mohsen Ebrahimi, Amir Ahmad Salarian, Seyyed Reza Abtahi, Alireza Jahandideh

**Affiliations:** 1 *Faculty of Medicine, AJA University of medical sciences, Tehran, Iran*; 2 *Department of Toxicology and Pharmacology, Faculty of Medicine, AJA University of Medical Sciences, Tehran, Iran*; 3 *Department of Clinical Science, Faculty of Specialized Veterinary Sciences, Science and Research Branch, Islamic Azad University, Tehran, Iran*

**Keywords:** Methicillin-resistant staphylococcus aureus (MRSA), *Crocus pallasii subsp. haussknechtii boiss*, Diabetes, Wound healing

## Abstract

**Objective:**

To evaluate the wound healing activity of *Crocus pallasii subsp. haussknechtii boiss* leaves extract on infected wounds in diabetic rats.

**Methods:**

Fifty male diabetic rats were randomized into two sets of 25 animals each. Each group was sub divided into five groups of five animals, each for excisional and incisional wound models, respectively. Induction of diabetes was achieved using 60 mg/kg streptozotocin. In group I, 0.1 mL sterile saline 0.9% solution was added to the wounds with no infection. In group II, the wounds were infected with Methicillin-resistant staphylococcus aureus (MRSA) and only treated with 0.1 mL the sterile saline 0.9% solution. In group III, infected wounds were treated with application of base formulation ointment. In group IV, animals with infected wounds were treated with 0.1 mL topical application of 1 mg/mL methicillin and base formulation ointment. In group V, animals with infected wounds were treated with topical application of 0.1 mL solution of methicillin (1 mg/mL) and with 1g of powder extract of the plant material in ointment. The healing of the wound was assessed based on planimetry, hydroxyproline estimation, microbiological, biomechanical and biochemical studies

**Results:**

Microbiological examination, planimetric, histological and quantitative morphometric studies and determination of hydroxyproline levels showed that there was significant difference between animals in group V compared to other groups (*p*=0.001). Biomechanical indices in incisional groups showed there was significant difference between animals in group V compared to other groups (*p*=0.001).

**Conclusion:**

It was possible to conclude that the ointment of the extract of *Crocus pallasii subsp. haussknechtii boiss*. leaves have significant wound-healing activity in diabetes.

## Introduction

The prevalence of diabetes has become a major clinical problem and a serious issue for public health. The impaired wound healing in diabetic patients is one of the complications [1]. Lack of cellular and molecular signals required for normal wound repair process such as angiogenesis, granulation tissue formation, epithelialization, and remodeling are encountered in diabetic patients that contribute to the poor healing of diabetic wound. The normal healing process in healthy individuals occur at an optimal rate, however, it is usually delayed or even completely compromised in diabetic patients [1].

In diabetes wound healing is impaired that could be associated with neuropathy, vascular disease and foot deformities [2]. At the level of the cells, a surge in the number of acute inflammatory cells, lack of cellular growth and migration of the epidermis have been observed [3]. Patients with diabetes have impaired leukocyte function and the metabolic abnormalities of diabetes ends up impaired migration of neutrophils and macrophages to the wound as well as reduced chemotaxis [4]. These changes at cellular levels would predispose individuals to an increased risk of wound infection. In diabetes, impaired wound healing and other tissue abnormalities are considered as major concerns [5]. The biochemical mechanisms involved in the healing process are mainly associated with disorders in collagen production that consequently end up delayed re-epithelialization in wounds, compromised migration and proliferation of keratinocytes and fibroblasts [6]. Various treatments have been adopted to solve this complex clinical problem, however, only a few have been proven to be effective [7]. 

There is an increasing interest in the potential of traditional and complementary medicines to be used in in the area of wound that has led to investigations of a range of plant extracts and other products as traditionally wound healing agents [8]. These agents usually influence one or more phases of the healing process, and are also involved in disinfection and provide a moist environment to encourage the establishment of a suitable environment for the natural healing process [8].

The plant extracts with wound healing properties have the potential for antioxidant, chelation and antimicrobial activities; and may act by one or more of these mechanisms [9]. Natural antioxidants have been reported to play a major role in blocking the oxidative stress induced by free radicals [10]. Therefore, it is very important to find out new sources of safe, and in extensive antioxidants of natural origin. Recently, many researchers have shown interest in edible and medicinal plants for their phenolic contents and related total antioxidant activities [11,12].

Wild edible plants with antioxidant activities are important constituents of traditional diets in Iran. Some of these plants have not been screened for their wound healing potentials. This information is necessary to validate the safety uses of traditional plants and may be used to establish databases and find out new natural remedies with wound healing potentials.

It has been demonstrated that *Crocus pallasii subsp. haussknechtii boiss.*leaves bear antioxidant properties using 1,1-diphenyl-2-picrylhydrazyl, ferric reducing ability of plasma, ferric reducing power, inhibition of lipid peroxidation, Trolox equivalent antioxidant capacity, total flavonoid content and total phenolic content methods. The antibacterial properties of the *Crocus pallasii subsp. haussknechtii boiss. *leaves have also been approved using disk diffusion method measured by diameter of inhibition zones [13].

A literature survey reveals that no systematic approach has been made to study the wound-healing activity of the extract of *Crocus pallasii subsp. haussknechtii boiss.*leaves. In the present work, the wound-healing activities of the methanolic extract of *Crocus pallasii subsp. haussknechtii boiss.* leaves were investigated in an ointment form in infected diabetic wounds. Assessment of the healing process was based on planimetry, hydroxyproline estimation, microbiological, biomechanical and biochemical studies.

## Materials and Methods

Our study protocol was reviewed and approved by the Ethical Committee of AJA University of medical Sciences under (No. ECAJAUMS/997953-15-11-1397). All animals received humane care in accordance with the Guide for the Care and Use of Laboratory Animals prepared by the National Academy of Sciences and published by the National Institutes of Health (NIH publication No. 85–23, revised 1985). 

*Plant material and extract preparation*

The plant samples were collected from the Western provinces of Iran in April and May 2018. Specimens from the plant material were deposited and authenticated at the Department of Botany Sciences, the Hamadan Research Agricultural and Natural Rescores Center, Hamadan, Iran. The leaves of the plant were used in the study. The plant material crisp was powdered in an electric blender. For the methanolic extraction, 150 g of the fine powder was extracted with 600 mL of 80 % methanol at 37 ºC for 3 hours. The sample was then centrifuged at 4500 rpm for 15 minutes and the supernatants were used. The filtrate was placed in an oven to dry at 40 ºC. The obtained clear residue was used for the study. Ash and moisture contents were determined based on a method described by others [14]. In brief, 3 g of leaves was incinerated in a Silica crucible over the burner. The charred material was heated in muffle furnace for six hours at 600-650 °C. The ash was cooled and weighed on the filter paper. The Moisture was determined by drying the sample at 105°C. The moisture content was expressed as a percent of the oven dry mass or of the as-received mass. 

*Phenolic contents, flavonoid contents and antioxidant activity of methanolic extract of Crocus pallasii subsp. haussknechtii boiss. leaves*

The radical scavenging capacity of the extract (DPPH) was estimated using the Brand-Williams method [15]. The ferric reducing ability of plasma (FRAP) assay was performed based on a method described by Benzie and Strain with a slight modification [16]. The total phenolic content (TPL) of the extract was determined using the Folin-Ciocalteu reagent based on a procedure described by others [17]. The total phenolic content was expressed as gallic acid equivalents in mg/g of chloroform extract. The Trolox equivalent antioxidant capacity (TEAC) assay was based on the ability of the extract to scavenge the stable ABTS radicals [18]. The sample was mixed with 3.0 mL of ABTS solution and the absorbance was then measured at 734 nm. For the lipid peroxidation inhibition assay (LPI), the extract (40 mL) was mixed with 4.1 mL linoleic acid in absolute ethanol (2.51%), 8 mL of phosphate buffered (pH = 7.0; 0.05 M) and 3.9 mL of distilled water. The mixture was then placed in an oven at 40 ºC. Then, 70% ethanol (9.7 mL) and 30% ammonium thiocyanate (0.1 mL) were added to the mixture (0.1 mL). Three min after addition of 0.1 mL of ferrous chloride (0.02M in 3.5% hydrochloric acid) to the reaction mixture, the absorbance of red color was measured at 500 nm [19]. The total flavonoid content (TF) of the extract was measured using a method described by others [20]. The ferric reducing power (FRP) of the extract was measured based on the method of the others [21].

*Formulation of the ointment*

The base formulation consisting of Eucerin (30%) and Vaseline (70%) in about 1: 2 proportions were prepared. The topical application form was prepared comprising 2g powder extract of the plant material in 98 g ointment.

*Sample size formula*

A power calculation based on earlier studies suggested that fifty male diabetic rats with two sets of 25 animals for each of excisional and incisional wound models would be sufficient to detect a statistically significant difference for the analyses, which was the primary outcome in this study [22]. 

*Induction of diabetes*

Animal houses were in standard environmental conditions of temperature (22 ±3°C), humidity (60 ± 5%), and a 12h light/dark cycle. The animals were maintained on standard pellet diet and tap water. The animals were Wistar, male, healthy, approximately 280 g and 7 weeks of age. Induction of diabetes was based on a method described by others [5]. Briefly, for insulin-deficient diabetes, rats were fasted overnight before receiving a single intraperitoneal injection (50 mg/kg in 0.9% sterile saline) of streptozotocin (STZ). Hyperglycemia (15 mmol/l or greater) was confirmed 2 days later by measurement of tail-vein blood glucose concentration using a glucometer (Ames Glucostix; Myles, Elkhart, IN). The rats underwent the procedures three days after induction of diabetes. The rats were approximately 250 g following induction of diabetes.

*Excision wound model and planimetric studies*

Rats were anesthetized by an intraperitoneal injection of ketamine-xylazine (ketamine 5%, 70 mg/kg and xylazine 2%, 5mg/kg, Alfasan, the Netherlands). The hair on their back was shaved and the skin cleansed with 70% alcohol solution. Following shaving and aseptic preparation, a circular excision wound was made by cutting away approximately 115 mm^2^ full thickness of predetermined area on the anterior-dorsal side of each rat. Small gauze was placed over each wound and then inoculated with 5 × 10^7^ CFU of *Staphylococcus aureus *ATCC 43300. The methicillin-resistant *S. aureus *ATCC 43300 strain was commercially available. The pocket was closed by means of 4-0 nylon sutures (Ethicon,Ltd, U.K.) and this procedure resulted in a local abscess after 24 h. The rats were returned to individual cages and they were examined daily. After 24 h, the wounds were opened, the gauze removed for quantitative bacterial cultures and treatment started. 

For excisional wound healing model 25 healthy male Wistar rats weighing approximately 160-180 g and seven weeks of age were randomized into five groups of five animals each. Induction of diabetes was achieved using 60 mg/kg streptozotocin. In group I, 0.1 mL sterile saline 0.9% solution was added to the wounds with no infection. In group II, the wounds were infected with MRSA and only treated with 0.1 mL the sterile saline 0.9% solution. In group III, infected wounds were treated with application of base formulation ointment. In group IV, animals with infected wounds were treated with 0.1 mL topical application of 1 mg/mL methicillin and base formulation ointment. In group V, animals with infected wounds were treated with topical application of 0.1 mL solution of methicillin (1 mg/mL) and with 1g of powder extract of the plant material in ointment. All the test formulations were applied for 10 days starting from the day of wounding. Wound-healing property was evaluated by wound contraction percentage and wound closure time. Photographs were taken immediately after wounding and on days 0, 7, 14 and 21 post-operation by a digital camera while a ruler was placed near the wounds. The wound areas were analyzed by Measuring Tool of Adobe Acrobat 9 Pro Extended software (Adobe Systems Inc, San Jose, CA, USA). Wound closure formula was based on other studies [22]: 

Percentage of wound contraction = (A_0_ – A_t_) / A_0_ × 100 

Where A_0_ is the original wound area and A_t_ is the wound area at the time of imaging. The animals were left in separate cages for four days at room conditions for acclimatization

*Microbiological assessments*

Briefly, for total bacterial count on days 7 and 14 of treatment after wound creation the granulated tissues were excised aseptically with animals under anesthesia. Then, 0.1 g of samples were crushed and homogenized in sterile mortar containing 10 ml of sterile saline. The homogenized samples were serially diluted in tube containing 9 ml of sterile saline to 10-5. The diluted samples were cultured on plate count agar (Merck KGaA, Darmstadt, Germany) superficially and duplicated. The cultured plates were incubated at 37 ºC for 24 to 48 hours. After incubation, all colonies were counted and results described as CFU/g of granulation tissue.

*Determination of hydroxyproline levels*

On the day 21 after surgery, a piece of skin from the healed wound area was collected and analyzed for hydroxyproline content. The animals were then euthanized with overdose of Ketamine-Xylazine combination. As a major part of collagen, hydroxyproline has an essential role in collagen stability. The collagen is the major component of extracellular tissue, which gives support and strength. The hydroxyproline contents were estimated using a method described by others [20]. Briefly, tissues were dried in a hot air oven at 60–70 ◦C to constant weight and were hydrolyzed in 6N HCl at 130 ◦C for 4 h in sealed tubes. The hydrolysate was neutralized to pH 7.0 and was subjected to chloramine-T oxidation for 20 min. The reaction was terminated by addition of 0.4M perchloric acid and color was developed with the help of Ehrlich reagent at 60 ºC and measured at 557 nm using UV-visible spectrophotometer (CamSpec M330, Cambridge CB2 4BG, UK).

*Incision wound model and biomechanical testing*

Another twenty-five diabetic rats were randomized into five groups of five rats each. All animals of five groups were grouped and anesthetized as mentioned above and a paravertebral long incision of 4 cm length was made through the skin and cutaneous muscle at a distance about 1.5 cm from the middle on right side of the depilated back. After the incision was made, the two sides of the wound were sutured at 0.5 cm intervals with 3/0 nylon suture material. The formulations were applied the same way in the excisional wound model. Ointments were applied once daily for 9 days. On day 9, sutures were removed and a strip of skin, 7 cm long, with the same widths of wound diameter, in the manner that the wound was located at the middle of the strip, was removed by a double-blade scalpel. The skin was then wrapped in Ringer’s soaked gauze, aluminum foils, and plastic bags and kept in −20°C freezer until mechanical testing. The TA.XTPlus Texture Analyzer mechanical test device was used for the assessment (Stable Micro Systems, Surrey GU7 1YL, UK). The samples were fitted with appropriate clamps, the distance between the clamps at the start of testing being 4 cm. The strips were loaded with 0–30 kg load cell, with strain rate of 1 cm/min and the load elongation curves were obtained. Yield strength (yield point) (kg), ultimate strength (kg), maximum stored energy (kg/cm), and stiffness (kg/cm) were measured from the load elongation curves. 

*Histological preparation and quantitative morphometric studies*

The histopathological criteria were based on a method described by others [23]. The tissue samples were taken on 7, 14, 21 days after surgery from periphery of the wound along with normal skin in excisional wound model and fixed in 10% buffered formalin, drhydrated and embedded in paraffin wax, sectioned at 5 µm and stained with hematoxylin and eosin (H&E) and Masson’s trichrome stains. Photomicrographs were obtained under light microscope to assess the predominant stage of wound healing. Three parallel sections were obtained from each specimen. Cellular infiltration including the number of mononuclear cells, poly-morphonuclear cells and fibroblastic aggregation were quantitatively evaluated. Acute hemorrhage, congestion, vascularization, epithelialization, collagen production and density were also evaluated qualitatively. Morphological findings were scored using image analyzing software (Image-Pro Express, version 6.0.0.319, Media Cybernetics, Silver Springs, MD, USA). The histological parameters were classified according to the intensity of occurrence in five levels (- absence; + discrete; ++ moderate; +++ intense; ++++ very intense) [23].

*Biochemical analyses*

The frozen samples at -80 °C were homogenized in phosphate buffered saline and centrifuged at 5 °C. The supernatant was used for analysis of malondialdehyde (MDA), superoxide dismutase (SOD), glutathione S-transferase (GST) and Carbonyl proteins. GST and SOD analyses were performed based on methods described by others [24,25]. Carbonyl proteins were performed based on a protocol described by others [26]. The biochemical data were quantified based on the Bradford method and normalized in relation to total protein levels in the supernatant [27].

*Statistical analysis*

Differences among groups in hydroxyproline levels were evaluated by Kruskal–Wallis variance analysis. When the P-value from the Kruskal–Wallis test statistics was statistically significant, multiple comparison tests were used to know the differences. Student’s t-test was used for evaluation of other test results. For wound closure are repeated measurement test were adopted. Comparison among days was assessed by Mann–Whitney U-test. The Bonferroni correction was applied for all possible multiple comparisons. SPSS 11.5 (SPSS Inc., Chicago, IL, USA) was used for statistical analysis. A *P*-value was set at 0.05.

## Results

*Plant material, Phenolic contents, flavonoid contents and antioxidant activity*

The values for moisture and ash were 75.7 ± 2.5 and 22.8 ± 0.3, respectively. The methanolic extract of *Crocus pallasii subsp. haussknechtii boiss. *leaves demonstrated total phenolic content (55.25± 0.67 mg of gallic acid equivalents/g of dry weight), total flavonoid content (27.11 ± 0.33 mg catechin equivalents /g of dry weight), antioxidant activity using the FRAP assay 425.0 ± 3.37 µmol *Fe *(II)/g of dry weight), antioxidant activity using the ABTS assay (713.0 ± 9.55 µmol Trolox equivalent/g of dry weight), DPPH radical scavenging activity (85.44 ± 2.05%), reducing power (710.0 ± 6.80 µmol Trolox equivalents/g of dry weight) and inhibition against lipid peroxidation (73.40 ± 2.07%).

*Microbiological assessments*

In animals of group V whose infected wounds were treated with both methicillin and the extract, the counts of *S. aureus* cultured in the wound tissues were significantly lower than in the infected wounds in other groups (*p*=0.001).

No animals died due to infection or anesthetics. The uninfected wounds treated with saline had no CFU/g of *S. aureus* count. Topical application of 0.1 mL solution of methicillin (1 mg/mL) and the extract significantly reduced the rate of total bacterial count on 7 and 14 days post-wounding compared to other groups with infected wounds (*p*=0.001) (Table 1). 

*Reduction in wound area*

**Fig. 1:**
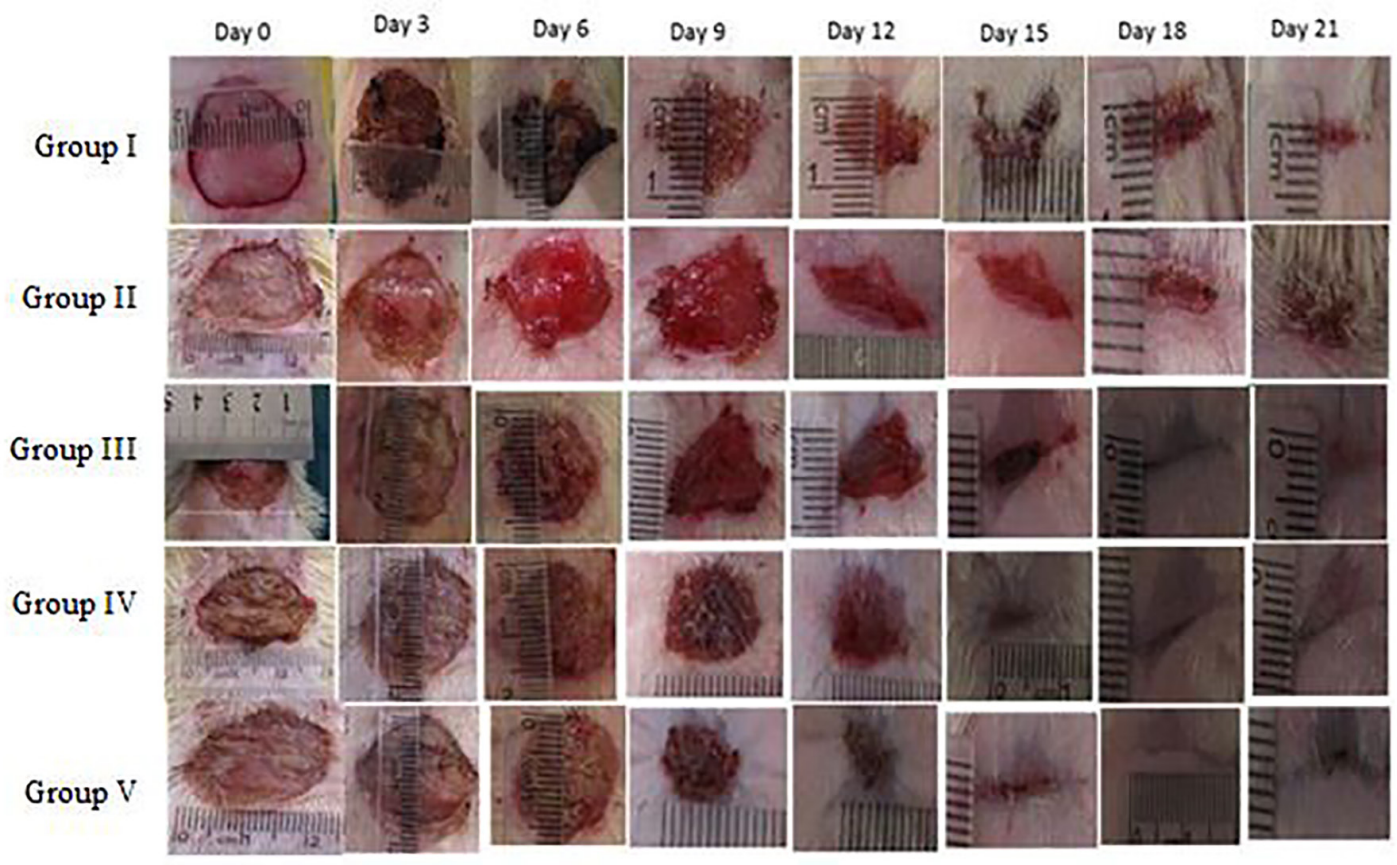
Serial representative photographs of the wounds from day 0 to day 21 in experimental groups

Wound contraction percentage in different groups during the course of study is shown in Table 2. The healing rate of the extract treated groups was significantly different compared to the control group (*p*= 0.001). However, time had significant effect on wound contraction of all wounds (*p*=0.037) (Figure 1).

*Hydroxyproline content of the wounds*

Proline is hydroxylated to form hydroxyproline after protein synthesis. Hydroxyproline contents in the groups I to V were found to be 81.17±2.99, 40.11±3.17, 66.44±4.57, 55.70±4.77 and 67.20±3.78 mg/g, respectively. Hydroxyproline contents were increased significantly in the extract treated group which implies more collagen deposition compared to infected wounds in other groups (*p*=0.001).

*Incision wound model and biomechanical testing*

The biomechanical indices, maximum stored energy, stiffness, ultimate strength and yield strength obtained for the extract treated groups were significantly higher than those obtained for groups II to IV (*p*=0.003) (Table 3). 

*Histological and morphometric findings*

**Fig. 2:**
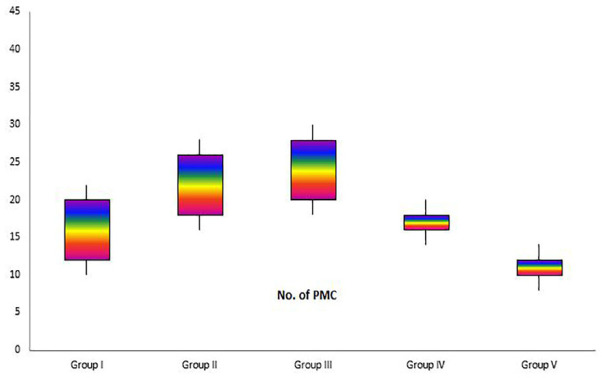
Box-plot indicating number of polymorphnuclear cells (PMN) in excisional model of the rat skin in experimental groups

**Fig. 3:**
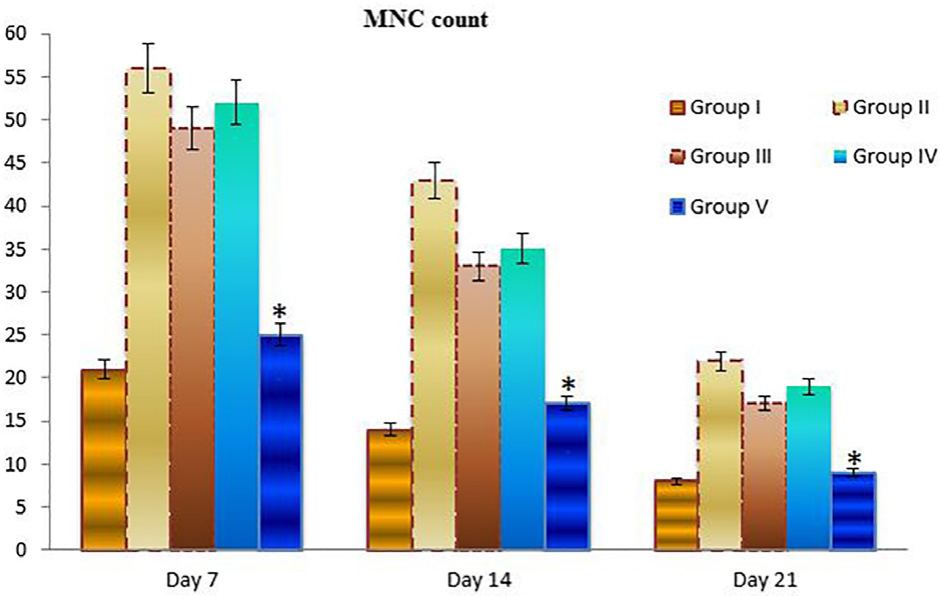
Bar graph indicating number of mononuclear cells (MNC) in excisional model of the rat skin in experimental groups. Results were expressed as mean ± SEM. * *P* < 0.05 vs. other experimental groups

**Fig. 4:**
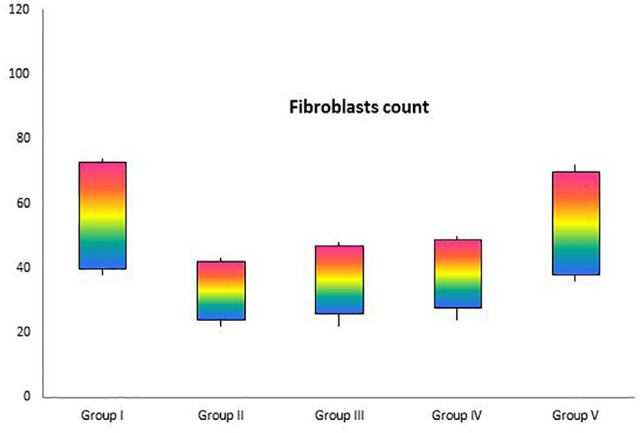
Box-plot indicating number of fibroblasts in excisional model of the rat skin in experimental groups

**Fig. 5:**
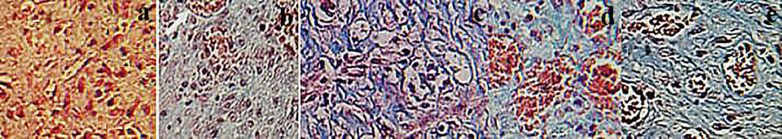
Representative micrographs of histological characteristics of rat skin on day 14 after wound creation in excisional wound model. a: Group I, b: Group II, c: Group III, d: Group IV and e: Group V. Masson trichrome staining (×400)

There were significant differences in comparisons of the extract treated and non-extract treated groups, particularly in terms of cellular infiltration, acute hemorrhage, congestion, edema, collagen production and density, re-epithelialization and neovascularization. During the study period, scores for reepithelialisation and neovascularization were significantly higher in animals of the extract treated groups than infected wounds in other groups (*p*=0.001). Polymorphonuclear (PMN) and mononuclear (MNC) cell count, fibroblast cell proliferation and also Mean Rank of the qualitative study of acute hemorrhage, edema and collagen production score in the extract treated groups were significantly higher than those infected wounds in other groups (*p*=0.001) (Table 4) (Figures 2, 3, 4 and 5).

*Biochemical findings*

The MDA values were significantly reduced in the extract treated groups in comparison with infected wounds in other groups on day 14 (*p*=0.0032). On 21, the MDA levels in extract treated group were significantly decreased compared to infected wounds in other groups (*p*=0.001). The SOD levels were significantly higher in the extract treated animals compared to infected wounds in other groups (*p*=0.001). The SOD levels in the extract treated group were significantly decreased compared to infected wounds in other groups on day 14 (*p*=0.0034). There were no significant differences in GST levels among groups during the study period (*p*=0.075). Carbonyl proteins were significantly lower in the extract treated animals compared to extract treated (Table 5).

## Discussion

Indigenous and traditional medicines make extensive use of natural products and derivatives of natural products and provide more than half of all medicines consumed today throughout the world. Recognizing the important role traditional medicine continues to play, extensive survey of literature reporting the use of medical plants and plant-based products for cutaneous wounds have been reported [28].

Wound healing is characterized by reepithelialization, granulation tissue growth and remodeling of extracellular matrix. Although the wound healing process occurs by itself, spontaneously, and does not require much help, there are various risk factors such as infection, supply of blood, nutritional status and other factors that influence the resolution of this process [29]. It is well known that attack by microbes, which invade the skin barrier, delays the natural wound healing process [30]. MRSA is increasing in infections and is a serious threat to patients in health care facilities and the community. There are many reports in the literature that researchers have been working on various agents to combat MRSA related infections [31,32]. Resistance to common antibiotics makes treating MRSA costly and difficult. The main end point observed in this study, wound contraction and reduction in wound area, was accelerated by treating the wounds with methicillin in presence of extract of *Crocus pallasii subsp. haussknechtii boiss*. leaves. All the parameters observed (presence of necrotic tissue, clotting and crust, re-epithelialization and granulation tissue growth, bacterial count) were affected; suggesting that methicillin in presence of extract of *Crocus pallasii subsp. haussknechtii boiss*. leaves were effective against MRSA. Topical application of extract of *Crocus pallasii subsp. haussknechtii boiss*. leaves at the wound site produced significant wound healing activity, indicating that it could have sensitized MRSA to methicillin that needs in depth and more extended works.

In excisional wound model there was a significant decrease in wound area. This indicated improved collagen maturation by increased cross linking. The balance between synthesis and breakdown and so deposition of collagen is important in wound healing and development of wound strength [33]. Collagen, the major component which strengthens and supports extra cellular tissue, is composed of the amino acid hydroxyproline, which has been used as a biochemical marker for tissue collagen [34]. In excisional wound model in the extract treated animals there was a significant decrease in wound area. This indicated improved collagen maturation by increased cross linking. The balance between synthesis and breakdown and so deposition of collagen is important in wound healing and development of wound strength [35]. Hydroxyproline is a major component of the collagen that permits the sharp twisting of the collagen helix. It helps on providing stability to the triple-helical structure of collagen by forming hydrogen bonds. Hydroxyproline is found in few proteins other than collagen. For this reason, hydroxyproline content has been used as an indicator to determine collagen content [36]. Increase in hydroxyproline content in the extract treated groups indicated increased collagen content, since hydroxyproline is the direct estimate of collagen synthesis. 

Mechanical testing is sensitive to changes that occur during the progression of wound healing, and can be used as a tool to measure the quality of healing. Mechanical property data provide a clinically relevant and functional assessment of wound healing quality. Histological analyses highlight cellular and connective tissue adaptation at the ultra-structural level in the repair process [37]. When compared to other experimental groups, the extract treated animals showed a statistically significant difference in biomechanical parameters.

In the present study, histopathological examination and scoring revealed that there was a significant difference by means of wound healing scores in the extract treated groups compared to other experimental groups. The extract decreased the maturation time of granulation tissue and wound contraction which means that it enhanced reepithelialisation with significant effect on inflammatory infiltration and number of fibroblasts in time-dependent activity. 

Antioxidants have been reported to play a significant role in improving the wound-healing process and protecting the tissues from oxidative damage [38]. Wound-healing mechanisms may be contributed to stimulate the production of antioxidants in wound site and to provide a favorable environment for tissue healing [38]. Increased oxidative damage might directly interfere skin tissue repair [31]. In the present study, the extract treated animals showed a significant increase in SOD levels compared to other groups, demonstrating the antioxidant effect of the extract in all concentrations. It could be concluding that the antioxidant effect of the extract provided an important anti-inflammatory response which could be associated with stimulation of antioxidant enzymes, particularly SOD, which remained at high levels in the extract treated animals. Furthermore, lower levels of MDA and carbonylated proteins were observed in the extract treated animals which are important markers of tissue stress.

Natural antioxidants have been reported to play a major role in blocking the oxidative stress induced by free radicals [39]. Therefore, it is very important to find out new sources of safe, and inexpensive antioxidants of natural origin. Recently, many researchers have shown interest in edible and medicinal plants for their phenolic contents and related total antioxidant activities [40]. Wild edible plants are important constituents of traditional diets in Iran. Some of these plants have not been screened for their antioxidant activity [41]. This information is necessary to validate the safety, and traditional uses of the plants, and may be used to establish antioxidant databases and find out new natural antioxidants [13]. The findings of the present study demonstrated that the extract of *Crocus pallasii subsp. haussknechtii boiss*. leaves were found to be promising in management of infected diabetic wound in rats.

Although the present study showed the promising effect of the extract of *Crocus pallasii subsp. haussknechtii boiss*. leaves on wound healing in diabetic rats, data regarding the molecular mechanisms leading to its action remain to be investigated in depth. The authors did not provide molecular evidence for the action of the extract. The dose-response studies were also needed to achieve maximum effect for the extract. These could be considered limitations of the present study.

The present study demonstrated that methanolic extract of *Crocus pallasii subsp. haussknechtii boiss*. leaves had properties that render it capable of promoting accelerated wound-healing activity compared to the controls. On the basis of the results obtained in the present study, it is possible to conclude that the ointment of the extract of *Crocus pallasii subsp. haussknechtii boiss*. leaves have significant wound-healing activity in diabetes.

**Table 1:** Wound bacterial count in experimental groups on tow time points of day 7 and day 14

**Groups**	**Wound bacterial count (CFU** ^a^ **/g) of granulation tissue**	
	**On day seventh**	**On day fourteenth**
**Group I**	0.00 ± 0.00	0.00 ± 0.00
**Group II**	1290.32 ± 260.10	1070.81 ± 220.19
**Group III**	1270.55 ± 220.85	980.65 ± 220.37
**Group IV**	1030.52 ± 280.27	970.34 ± 270.54
**Group V**	381.19 ± 40.25^b^	181.14 ± 47.21*

^a^ **CFU:** Colony-forming units; ^b^*P*<0.05 *vs*. groups III and IV.

**Table 2:** Area (mm^2^) in animals of experimental groups. Values are given as mean ± SEM

**Groups**	**Day 0**	**Day 7**	**Day 14**	**Day 21**
**Group I**	114.45±2.0	80.30±2.15	62.71±2.60	4.32±1.70
**Group II**	115.70±1.30	91.20±2.15	81.30±2.48	60.72±1.84
**Group III**	115.32±2.40	88.37±2.15	79.43±2.03	59.32±1.71
**Group IV**	114.50±2.50	76.68±2.50	69.17±2.72	50.93±1.48
**Group V**	115.03±2.10	62.55±2.25^a^	42.85±2.68^ a^	33.75±1.84^ a^

The treated groups are compared by Student t test with other groups; ^a^: The mean difference is significant at the .05 level *vs* II - IV. (Kruskal–Wallis test).

**Table 3:** Biomechanical indices assessed for each of the experimental groups. Values are given as mean ± SEM

**Biomechanical Indices**				
**Groups**	**Stiffness (Kg/cm)**	**Ultimate Strength (Kg)**	**Yield Point (Kg)**	**MES** ^a^ ** (Kg/cm)**
**Group I**	1.32 ± 0.12	1.17 ± 0.18	1.12 ± 0.24	1.33 ± 0.15
**Group II**	0.47 ± 0.15	0.77 ± 0.12	0.62 ± 0.11	0.65 ± 0.16
**Group III**	0.67 ± 0.10	0.81 ± 0.12	0.70 ± 0.19	0.92 ± 0.28
**Group IV**	0.98 ± 0.10	0.94 ± 0.17	0.78 ± 0.18	1.12 ± 0.24
**Group V**	1.69 ± 0.17^b^	1.55 ± 0.12^ b^	1.48 ± 0.19^ b^	1.77 ± 0.35^ b^

^a^MES: Maximum Stored Energy; ^b^The mean difference is significant at the .05 level vs Grpous II-IV

**Table 4.** Evaluation of Intensity of histological parameters in experimental groups

**Group V**			**Group IV**			**Group III**			**Group II**			**Group I**			Histological Parameters
Day 21	Day 14	Day 7	Day 21	Day 14	Day 7	Day 21	Day 14	Day 7	Day 21	Day 14	Day 7	Day 21	Day 14	Day 7	
0*	0*	1*	1	2	3	0	1	2	2	3	4	0	2	3	**Acute Hemorrhage**
0*	0*	1*	1	2	3	2	1	2	2	3	3	0	1	3	**Congestion**
4*	4*	3*	2	2	1	3	3	3	1	1	0	3	2	1	**Vascularization**
4*	3*	2*	2	1	0	2	2	1	1	1	0	2	1	0	**Epithelialization**
4*	4*	2*	1	1	0	2	2	1	1	1	0	2	2	1	**Collagen**

Classification of histological parameters according to the intensity of occurrence: 0 absence; 1 discrete; 2 moderate; 3 intense; 4 very intense. Histopatological damages were assessed as explained under material and methods on days, 7, 14 and 21 of lesion.*P<0.05 *vs*. groups III and IV.

**Table 5:** Comparison of the activities of MDA, SOD, GST and carbonyl proteins in the tissue samples taken from experimental groups on days 7, 14 and 21. Data are expressed as Mean ± SD

**Variables**	**Group I**	**Group II**	**Group III**	**Group IV**	**Group V**
**Day** ** 7**					
**MDA** ^a^ (nmol/mg protein)	0.070 ± 0.015	0.065 ± 0.017	0.062 ± 0.015*	0.064 ± 0.013	0.067 ± 0.015^ d^
**SOD** ^b^ (U/mg protein)	0.25 ± 0.07	0.83 ± 0.27	7.85 ± 0.15	7.97 ± 0.11	5.86 ± 0.14^ d^
**GST** ^c^ (µmol/min/g)	0.43 ± 0.17	0.48 ± 0.15	0.49 ± 0.12	0.44 ± 0.15	0.43 ± 0.17
Carbonyl Proteins (nmol/mL)	73.40 ± 6.12	75.38 ± 5.55	64.30 ± 4.12	75.42 ± 4.55	22.81 ± 3.60^ d^
**Day 14**					
**MDA** ^a^ (nmol/mg protein)	0.080 ± 0.15	0.073 ± 0.011	0.064 ± 0.018	0.066 ± 0.014	0.059 ± 0.010^ d^
**SOD** ^b ^(U/mg protein)	0.63 ± 0.05	0.65 ± 0.04	0.63 ± 0.12	0.74 ± 0.14	1.20 ± 0.15^ d^
**GST** ^c^ (µmol/min/g)	0.48 ± 0.15	0.46 ±0.10	0.45 ± 0.15	0.45 ± 0.17	0.40 ± 0.12
Carbonyl Proteins (nmol/mL)	75.60± 4.80	73.69 ± 4.35	65.33 ± 4.40	67.88 ± 4.19	23.60 ± 4.70^ d^
**Day 21**					
**MDA** ^a ^(nmol/mg protein)	0.095 ± 0.023	0.084 ± 0.012	0.076 ± 0.010	0.078 ± 0.015	0.037 ± 0.015^ d^
**SOD** ^b^ (U/mg protein)	0.23 ± 0.05	0.22 ± 0.07	0.21 ± 0.15	0.23 ± 0.37	0.26 ± 0.23
**GST** ^c^ (µmol/min/g)	0.47 ± 0.15	0.45 ± 0.15	0.46 ± 0.18	0.44 ± 0.12	0.49 ± 0.18
Carbonyl Proteins (nmol/mL)	27.33 ± 4.75	25.78± 4.10	24.54 ± 3.20	21.70 ± 3.37	15.55 ± 3.38^ d^

^a^MDA: Malondialdehyde; ^b^SOD: Superoxide dismutase; ^c^GST: Glutathione S-transferase, ^d^ *P*<0.05 *vs.* groups IV and III.
